# Editorial: The Habenula and Its Role in Neuropsychiatric Symptoms

**DOI:** 10.3389/fnbeh.2022.929507

**Published:** 2022-05-24

**Authors:** Flavia Venetucci Gouveia, Phillip Michael Baker, Manuel Mameli, Jurgen Germann

**Affiliations:** ^1^Neuroscience and Mental Health, The Hospital for Sick Children Research Institute, Toronto, ON, Canada; ^2^Department of Psychology, Seattle Pacific University, Seattle, WA, United States; ^3^The Department of Fundamental Neuroscience, The University of Lausanne, Lausanne, Switzerland; ^4^INSERM, UMR-S 839, Paris, France; ^5^Division of Neurosurgery, Department of Surgery, University Health Network and University of Toronto, Toronto, ON, Canada

**Keywords:** habenula, psychiatric disorder, reward & motivation, monoaminergic system, neuroimaging, neuromodulation

The habenula (Hb) is a small epithalamic structure that, through its downstream connectivity controls major neurotransmitters, such as the cholinergic and monoaminergic systems (Hikosaka, 2010; Hu et al., 2020). The Hb contributes to a wide range of behaviors e.g. social behavior (van Kerkhof et al., 2013; Ogawa and Parhar, 2021), circadian rhythms (Liu et al., 2021; Salaberry and Mendoza, 2022), reward processing (Lalive et al., 2022; Mondoloni et al., 2022), decision-making (Stopper et al., 2014; Baker et al., 2015, 2017; Nuno-Perez et al., 2021), cognitive flexibility (Vadovičová, 2014; Baker et al., 2015, 2017), and is implicated in the neurobiology of a number of psychiatric disorders and neuropsychiatric symptoms (Hu et al., 2020). Recent findings confirmed the importance of the Hb in schizophrenia (Schafer et al., 2018; Li et al., 2019; Germann et al., 2020; Wang et al., 2020), bipolar disorder (Schafer et al., 2018; Zhang et al., 2019; Germann et al., 2020; Sonkusare et al., 2022), autism (Germann et al., 2021; Murru et al., 2021), depression (Yang et al., 2018; Barreiros et al., 2022; Zhang et al., 2022; Young et al.), and eating disorders (Maldonado et al., 2018; Wills et al., 2020; Carlson et al., 2022), and implicated the Hb in neuropsychiatric symptoms such as sleep disturbances (Aizawa et al., 2013; Ge et al., 2021), and agitation/aggressive behavior (Flanigan et al., 2017, 2020; Gan et al., 2019).

Thus, this timely special issue provided the space and opportunity for both clinical and pre-clinical researchers to have an up to date discussion of the important and broad role of the Hb in the various neuropsychiatric disorders and symptoms. In total, 60 authors from 8 different countries participated.

Emphasizing the broad role of the Hb, Baker et al. highlight some of the less explored aspects of lateral habenula (LHb) function in contextual memory, sleep, and behavioral flexibility, by providing evidence that the LHb is well-situated to integrate different internal states and multimodal sensory information. The authors focus on the impact of early life stress on LHb function to illustrate how dysregulations on LHb systems promote anhedonia and motivational deficits, and stress the importance of ethologically-relevant behaviors to further understand LHb involvement in a wide range of psychiatric illnesses. Illustrating the important role of the LHb in motivation Sevigny et al. using a unique behavioral paradigm that requires rats to climb a physical barrier in order to receive a large reinforcement or to opt for a smaller reward without the need to climb a barrier, show that pharmacological inactivation of the LHb results in fewer choices for the high-effort-high-reward option, demonstrating that the LHb is part of the circuit responsible for integrating external information on a trial-by-trial basis. This work points to the involvement of the LHb in the ability to discriminate rewards specifically when contingencies change in an unpredictable manner. This supports a growing body of experimental evidence arguing for a relevant contribution of the Hb in diverse facets of reward encoding (Stopper et al., 2014; Lalive et al., 2022).

Considering the important role of eye contact as the starting point of interactions in many social animals, Lee and Hikosaka recorded eye movement and LHb activity while monkeys viewed faces in the context of Pavlovian and instrumental conditioning tasks. The results show that faces associated with larger rewards elicited longer periods of eye contact and are associated with suppression of LHb neurons. Faces signaling low values are associated with excitation of LHb neurons. The authors conclude that the reward encoding of LHb contributes to social behavior and disorders, as a sequential goal-directed behavior. Webster et al., in their review, provide an up-to-date summary of the current state of knowledge on LHb neuronal activity and its association with Major Depressive Disorder (MDD). They discuss the growing body of literature on LHb excitatory and inhibitory neurons, downstream connections with the rostromedial tegmental nucleus, and involvement of the reward system, arguing that normalizing inhibitory signaling within the LHb may be a potential therapeutic strategy for MDD. Further studies are necessary to better understand the exact pharmacological and neural circuit mechanisms underlying inhibitory signaling within the LHb.

Another line of research associating Hb activity and MDD, involves circadian rhythms and light signals that affect the LHb. Young et al. review the literature regarding neuronal activity in the LHb during altered circadian rhythms and link it to mental disorders, including depression. The authors, however, highlight the need for further research before firm conclusions can be drawn regarding the importance of changes in the circadian function of the LHb in the etiology of depression and antidepressant treatments. New research by Elias et al. on the role of the Hb in the therapeutic effect of Deep Brain Stimulation (DBS) for MDD, showed clinical response to treatment was significantly associated with Hb volume changes, with responders showing increased Hb volume over time, and non-responders showing the opposite. Furthermore, functional MRI analysis showed DBS treatment to be significantly associated with increased Hb connectivity to several prefrontal and corticolimbic regions, areas previously implicated in the neurocircuitry of depression.

DBS targeting the Hb has been trialed for a number of psychiatric disorders as outlined in the review article by Germann et al. Merging the knowledge from pre-clinical and clinical observations, and using both the published literature as well as registered clinical trials the work highlights the important role of the Hb in mental health. The outcomes of the ongoing clinical trials for treating schizophrenia, depression, obsessive-compulsive disorder, and bipolar disorder will provide further knowledge that will be necessary to confirm the clinical benefit of this promising intervention. To investigate possible mechanisms of action of Hb-DBS, Zhang et al. explored the transient effects of Hb stimulation in patients with bipolar disorder and schizophrenia. Commonly elicited effects of stimulation were numbness, heart rate changes, pain, and involuntary movements and these showed a pattern suggesting a potential somatosensory organization of the Hb.

Expanding on the involvement of the Hb in psychiatric disorders, Lee and Goto in their perspective review hypothesize that an initially hypoactive Hb during childhood in individuals with Attention-Deficit-Hyperactivity-Disorder (ADHD) may undergo compensatory changes during development, priming the Hb to be hyperactive in response to stress exposure and thereby increasing vulnerability to MDD in adulthood. They suggest that the Hb is involved in the neural network of both MDD and ADHD, via direct and indirect connections with dopaminergic and serotonergic neurons in midbrain nuclei. Suggesting a role of the Hb in anxiety disorders, Liu et al. find that ovariectomized (OVX)-induced anxiety-like behavior is associated with increased LHb activity. Moreover, their results showed that estrogen-treated OVX rats present less anxiety-like behavior, higher levels of monoamines in dopaminergic and serotonergic nuclei, and reduced neuronal activity in the LHb, as compared to non-treated OVS rats. This effect is also observed following intra-LHb injections of estrogen receptor agonist in OVX rats. Gouveia and Ibrahim explore the anatomical organization of the Hb and discuss several distinct mechanisms by which the Hb is involved in the modulation of aggressive behaviors. They propose new investigations for the development of innovative neuromodulatory techniques targeting the Hb to reduce aggressive behaviors. Along those lines, Marks et al. propose that the LHb plays a critical role in the transition from suicidal ideations to self- harm. The authors argue that a multidisciplinary group of researchers is necessary to better understand the role of the LHb, and its long-term modulation, in response to the negative affect in suicidal behavior, to discern the underlying neural mechanisms of this contribution.

The studies presented in this special topic, highlight broad and important roles of the Hb in the neural-networks of several psychiatric disorders and neuropsychiatric symptoms, in both animal models and humans. This body of research points to the new experimental actions needed to further shed light on Hb cellular and molecular mechanisms, and its repercussions for physiological and pathological behaviors.

## Author Contributions

All authors listed have made a substantial, direct, and intellectual contribution to the work and approved it for publication.

## Conflict of Interest

The authors declare that the research was conducted in the absence of any commercial or financial relationships that could be construed as a potential conflict of interest.

## Publisher's Note

All claims expressed in this article are solely those of the authors and do not necessarily represent those of their affiliated organizations, or those of the publisher, the editors and the reviewers. Any product that may be evaluated in this article, or claim that may be made by its manufacturer, is not guaranteed or endorsed by the publisher.

## References

[B1] Aizawa H. Cui W. Tanaka K. Okamoto H. (2013). Hyperactivation of the habenula as a link between depression and sleep disturbance. Front. Hum. Neurosci. 7, 826. 10.3389/fnhum.2013.00826 24339810 PMC3857532

[B2] Baker P. M. Oh S. E. Kidder K. S. Mizumori S. J. Y. (2015). Ongoing behavioral state information signaled in the lateral habenula guides choice flexibility in freely moving rats. Front. Behav. Neurosci. 9, 295. 10.3389/fnbeh.2015.00295 26582981 PMC4631824

[B3] Baker P. M. Raynor S. A. Francis N. T. Mizumori S. J. Y. (2017). Lateral habenula integration of proactive and retroactive information mediates behavioral flexibility. Neuroscience 345, 89–98. 10.1016/j.neuroscience.2016.02.010 26876779

[B4] Barreiros A. R. Breukelaar I. Mayur P. Andepalli J. Tomimatsu Y. Funayama K.. (2022). Abnormal habenula functional connectivity characterizes treatment-resistant depression. Neuroimage. Clin. 34, 102990. 10.1016/j.nicl.2022.102990 35305499 PMC8933564

[B5] Carlson H. N. Christensen B. A. Pratt W. E. (2022). Stimulation of mu opioid, but not GABAergic, receptors of the lateral habenula alters free feeding in rats. Neurosci. Lett. 771, 136417. 10.1016/j.neulet.2021.136417 34954115

[B6] Flanigan M. Aleyasin H. Takahashi A. Golden S. A. Russo S. J. (2017). An emerging role for the lateral habenula in aggressive behavior. Pharmacol. Biochem. Behav. 162, 79–86. 10.1016/j.pbb.2017.05.003 28499809 PMC5659946

[B7] Flanigan M. E. Aleyasin H. Li L. Burnett C. J. Chan K. L. LeClair K. B.. (2020). Orexin signaling in GABAergic lateral habenula neurons modulates aggressive behavior in male mice. Nat. Neurosci. 23, 638–650. 10.1038/s41593-020-0617-7 32284606 PMC7195257

[B8] Gan G. Zilverstand A. Parvaz M. A. Preston-Campbell R. N. d'Oleire Uquillas F. Moeller S. J.. (2019). Habenula-prefrontal resting-state connectivity in reactive aggressive men - a pilot study. Neuropharmacology 156, 107396. 10.1016/j.neuropharm.2018.10.025 30366001 PMC6478575

[B9] Ge F. Mu P. Guo R. Cai L. Liu Z. Dong Y.. (2021). Chronic sleep fragmentation enhances habenula cholinergic neural activity. Mol. Psychiat. 26, 941–954. 10.1038/s41380-019-0419-z 30980042 PMC6790161

[B10] Germann J. Gouveia F. V. Brentani H. Bedford S. A. Tullo S. Chakravarty M. M.. (2021). Involvement of the habenula in the pathophysiology of autism spectrum disorder. Sci. Rep. 11, 21168. 10.1038/s41598-021-00603-0 34707133 PMC8551275

[B11] Germann J. Gouveia F. V. Martinez R. C. R. Zanetti M. V. de Souza Duran F. L. Chaim-Avancini T. M.. (2020). Fully automated habenula segmentation provides robust and reliable volume estimation across large magnetic resonance imaging datasets, suggesting intriguing developmental trajectories in psychiatric disease. Biol. Psychiat. Cogn. Neurosci. Neuroimag. 5, 923–929. 10.1016/j.bpsc.2020.01.004 32222276

[B12] Hikosaka O (2010). The habenula: from stress evasion to value-based decision-making. Nat. Rev. Neurosci. 11, 503–513. 10.1038/nrn2866 20559337 PMC3447364

[B13] Hu H. Cui Y. Yang Y. (2020). Circuits and functions of the lateral habenula in health and in disease. Nat. Rev. Neurosci. 21, 277–295. 10.1038/s41583-020-0292-4 32269316

[B14] Lalive A. L. Congiu M. Lewis C. Groos D. Clerke J. A. Tchenio A.. (2022). Synaptic inhibition in the lateral habenula shapes reward anticipation. Curr. Biol. 32, 1829–36.e4. 10.1016/j.cub.2022.02.035 35259343

[B15] Li J. Yang S. Liu X. Han Y. Li Y. Feng J.. (2019). Hypoactivity of the lateral habenula contributes to negative symptoms and cognitive dysfunction of schizophrenia in rats. Exp. Neurol. 318, 165–173. 10.1016/j.expneurol.2019.05.005 31082390

[B16] Liu H. Rastogi A. Narain P. Xu Q. Sabanovic M. Alhammadi A. D.. (2021). Blunted diurnal firing in lateral habenula projections to dorsal raphe nucleus and delayed photoentrainment in stress-susceptible mice. PLoS Biol. 19, e3000709. 10.1371/journal.pbio.3000709 33690628 PMC7984642

[B17] Maldonado M. Molfese D. L. Viswanath H. Curtis K. Jones A. Hayes T. G.. (2018). The habenula as a novel link between the homeostatic and hedonic pathways in cancer-associated weight loss: a pilot study. J. Cachexia Sarcopenia Muscle 9, 497–504. 10.1002/jcsm.12286 29575771 PMC5989751

[B18] Mondoloni S. Mameli M. Congiu M. (2022). Reward and aversion encoding in the lateral habenula for innate and learned behaviours. Transl. Psychiat. 12, 3. 10.1038/s41398-021-01774-0 35013094 PMC8748902

[B19] Murru L. Ponzoni L. Longatti A. Mazzoleni S. Giansante G. Bassani S.. (2021). Lateral habenula dysfunctions in Tm4sf2 mice model for neurodevelopmental disorder. Neurobiol. Dis. 148, 105189. 10.1016/j.nbd.2020.105189 33227491 PMC7840593

[B20] Nuno-Perez A. Trusel M. Lalive A. L. Congiu M. Gastaldo D. Tchenio A.. (2021). Stress undermines reward-guided cognitive performance through synaptic depression in the lateral habenula. Neuron 109, 947–956.e5. 10.1016/j.neuron.2021.01.008 33535028 PMC7980092

[B21] Ogawa S. Parhar I. S. (2021). Role of habenula in social and reproductive behaviors in fish: comparison with mammals. Front. Behav. Neurosci. 15, 818782. 10.3389/fnbeh.2021.818782 35221943 PMC8867168

[B22] Salaberry N. L. Mendoza J. (2022). The circadian clock in the mouse habenula is set by catecholamines. Cell Tissue Res. 387, 261–274. 10.1007/s00441-021-03557-x 34816282

[B23] Schafer M. Kim J.-W. Joseph J. Xu J. Frangou S. Doucet G. E. (2018). Imaging habenula volume in schizophrenia and bipolar disorder. Front. Psychiat. 9, 456. 10.3389/fpsyt.2018.00456 30319463 PMC6165901

[B24] Sonkusare S. Ding Q. Zhang Y. Wang L. Gong H. Mandali A.. (2022). Power signatures of habenular neuronal signals in patients with bipolar or unipolar depressive disorders correlate with their disease severity. Transl. Psychiatry 12, 72. 10.1038/s41398-022-01830-3 35194027 PMC8863838

[B25] Stopper C. M. Tse M. T. L. Montes D. R. Wiedman C. R. Floresco S. B. (2014). Overriding phasic dopamine signals redirects action selection during risk/reward decision making. Neuron 84, 177–189. 10.1016/j.neuron.2014.08.033 25220811

[B26] Vadovičová K (2014). Affective and cognitive prefrontal cortex projections to the lateral habenula in humans. Front. Hum. Neurosci. 8, 819. 10.3389/fnhum.2014.00819 25386128 PMC4209891

[B27] van Kerkhof L. W. M. Damsteegt R. Trezza V. Voorn P. Vanderschuren L. J. M. J. (2013). Functional integrity of the habenula is necessary for social play behaviour in rats. Eur. J. Neurosci. 38, 3465–3475. 10.1111/ejn.12353 24103016 PMC3836018

[B28] Wang Y. Zhang C. Zhang Y. Gong H. Li J. Jin H.. (2020). Habenula deep brain stimulation for intractable schizophrenia: a pilot study. Neurosurg. Focus 49, E9. 10.3171/2020.4.FOCUS20174 32610295

[B29] Wills K. E. Gosnell S. N. Curtis K. N. Velasquez K. Fowler J. C. Salas R. (2020). Altered habenula to locus coeruleus functional connectivity in past anorexia nervosa suggests correlation with suicidality: a pilot study. Eat. Weight Disord. 25, 1475–1480. 10.1007/s40519-019-00746-0 31376112 PMC6995421

[B30] Yang Y. Cui Y. Sang K. Dong Y. Ni Z. Ma S.. (2018). Ketamine blocks bursting in the lateral habenula to rapidly relieve depression. Nat. Publish. Group. 554, 317–322. 10.1038/nature25509 29446381

[B31] Zhang C. Kim S.-G. Li D. Zhang Y. Li Y. Husch A.. (2019). Habenula deep brain stimulation for refractory bipolar disorder. Brain Stimul. 12, 1298–1300. 10.1016/j.brs.2019.05.010 31103455

[B32] Zhang C. Zhang Y. Luo H. Xu X. Yuan T.-F. Li D.. (2022). Bilateral Habenula deep brain stimulation for treatment-resistant depression: clinical findings and electrophysiological features. Transl. Psychiatry 12, 52. 10.1038/s41398-022-01818-z 35115488 PMC8813927

